# Real-world insights into patients with advanced NSCLC and *MET* alterations

**DOI:** 10.1016/j.lungcan.2021.06.015

**Published:** 2021-07-16

**Authors:** Marisa Bittoni, James Chih-Hsin Yang, Jin-Yuan Shih, Nir Peled, Egbert F. Smit, D. Ross Camidge, Rajeswara Rao Arasada, Dina Oksen, Emmanuelle Boutmy, Christopher Stroh, Andreas Johne, David P. Carbone, Paul K. Paik

**Affiliations:** a James Comprehensive Cancer Center, The Ohio State University, Columbus, OH, USA; b Department of Medical Oncology, National Taiwan University Cancer Center, Taiwan; c Department of Internal Medicine, National Taiwan University Hospital, Taipei, Taiwan; d Oncology Department, Shaare Zedek Medical Center, Jerusalem, Israel; e Department of Thoracic Oncology, Netherlands Cancer Institute, Amsterdam, The Netherlands; f Medical Oncology Department, University of Colorado, Aurora, CO, USA; g Research and Development, Merck Healthcare KGaA, Darmstadt, Germany; h Thoracic Oncology Service, Memorial Sloan Kettering Cancer Center, New York, NY, USA; i Department of Medicine, Weill Cornell Medical College, New York, NY, USA

**Keywords:** Advanced NSCLC, *MET* exon 14, *MET* amplification, Treatment patterns, Real-world data, Outcomes, Biomarkers, Systemic treatments

## Abstract

**Objectives:**

To describe characteristics, treatment and outcomes of non-small cell lung cancer (NSCLC) patients with *MET* alterations (*MET* exon 14 [*MET*ex14] skipping or *MET* amplification [*MET*amp]) in real-world clinical care.

**Methods:**

This non-interventional cohort study used real-world data extracted from electronic medical records from academic oncology sites in Israel, The Netherlands, Taiwan, and the USA. Patients had confirmed diagnosis of advanced (Stage IIIB–IV) NSCLC harboring *MET* alterations (date of diagnosis = index date) between 1 Jan 2010 and 30 Sept 2018. Medical history was assessed prior to and at the index date (baseline period), and outcomes from first date of treatment to death, loss to follow-up, or end of study period.

**Results:**

A total of 117 patients were included (*MET*ex14 n = 70; *MET*amp n = 47); testing methods were heterogeneous. Concomitant oncogenic mutations were more common in the *MET*amp cohort than *MET*ex14. Patients in the *MET*ex14 cohort were older than those in *MET*amp, and a larger proportion were never smokers. Anticancer first-line therapies received by patients (*MET*ex14; *MET*amp) included chemotherapy only (44%; 41%), MET inhibitors (33%; 29%), immune checkpoint inhibitor (ICI) mono-(12%; 15%) and combination-therapy (8%; 3%). Second-line therapies included chemotherapy (35%; 30%) and MET inhibitors (30%; 39%). In the *MET*ex14 cohort, objective response rate (ORR) was generally low (first-line 28%; second-line 30%); no patients who received ICIs had a response. In the *MET*amp cohort, ORR was 36% in first-line and 22% in second-line. Median (95% confidence interval) overall survival from start of first-line therapy was 12.0 months (6.8, 19.2) in the *MET*ex14 cohort and 22.0 months (9.8, 31.2) in *MET*amp.

**Conclusions:**

Heterogeneous treatments reflect the changing landscape and availability of new treatments, as well as the high unmet medical need in older, *MET*ex14 patients who had more advanced disease at diagnosis. MET-targeted therapies could be beneficial in patients with these rare *MET* alterations.

## 1. Introduction

The receptor for hepatocyte growth factor (HGF), a tyrosine kinase that is encoded by the mesenchymal epithelial transition factor (*MET*) oncogene, has a crucial role in cancer growth, invasion and metastasis. Oncogenic *MET* alterations can act as a primary driver of tumorigenesis, with tumor dependence on MET signaling for cancer initiation and progression, a phenomenon that is called ‘oncogene addiction’. *MET* exon 14 (*MET*ex14) skipping alterations and *MET* amplification have been identified as alterations that can convert *MET* into a primary oncogenic driver [1].

*MET*ex14 skipping alterations and *MET* amplification occur in a subset of treatment-naïve patients with non-small cell lung cancer (NSCLC) [1-3]. *MET* alterations are generally observed across different NSCLC histology subtypes [1,4].

NSCLC harboring *MET*ex14 skipping alterations in about 8–15% also harbor concomitant *MET* amplifications [4]; however, *MET* amplification as a primary driver also occurs as a distinct oncogenic event in the absence of *MET*ex14 skipping. Targeted therapies are being developed to target NSCLC harboring *MET*ex14 skipping alterations or *MET* amplification, and available information indicates that both *MET* alterations are predictive markers in NSCLC, conferring sensitivity to MET inhibition [1,2,5]. The level of amplification potentially has an impact on the efficacy of targeted treatment options, with patients with highly amplified tumors showing an expectedly better response to systemic treatment [6]. Among patients with advanced NSCLC, both oncogenic MET activation as well as MET overexpression were shown to predict shorter survival [1,7].

The common standard of care in first-line therapy for NSCLC, without oncogenic drivers, involves use of platinum-based chemotherapy or immune checkpoint inhibitors (ICIs) either as single components or in combination, depending on the level of expression of programmed death-ligand 1 (PD-L1) or potential contraindications such as poor performance status, often found in patients of advanced age [8-12]. For patients advancing to second-line therapy, monotherapy with ICIs should be considered, if not previously given. Patients with oncogenic-driven NSCLC should receive targeted therapy where available [9,13], as the efficacy of standard therapies might be impaired in oncogene-addicted disease [14,15]. Reduced effectiveness of ICI monotherapy, chemotherapy or combinations has also been reported in patients with *MET*ex14 NSCLC [16-21]. Due to the importance of identifying these oncogenic drivers in patients with NSCLC, clinical guidelines advocate to perform molecular testing prior to the selection of NSCLC therapy [9,22].

Published literature on advanced NSCLC bearing selected *MET* alterations in real-world settings is sparse, as there is a lack of data to characterize patients due to the recent recognition of *MET*ex14 skipping alterations as an oncogenic driver mutation. Knowledge of the natural history of the disease and the clinical course of these patients is, therefore, currently limited. This study aims to comprehensively describe the biomarkers, demographics and clinical characteristics of these patients, as well as their treatment patterns and outcomes in real-world clinical care.

## 2. Materials and methods

### 2.1. Study design

This non-interventional retrospective cohort study extracted electronic medical record (EMR) data from academic oncology sites across Israel, The Netherlands, Taiwan, and the United States of America (USA). Informed consent was obtained for each participating site where required. Study-eligible patients were aged minimum 18 years old with advanced or metastatic NSCLC (Stage IIIB/IV) that was diagnosed between 1 January 2010 and 31 March 2019, and had a confirmed presence of *MET*ex14 skipping alterations/*MET* amplification at any time. The study was approved by the relevant Ethics Committees, Institutional Review Board, and/or local regulations of each site.

Two distinctive patient cohorts were studied: a *MET*ex14 cohort composed of patients harboring *MET*ex14 skipping alterations with or without concomitant *MET* amplification, and a *MET*-amplification cohort that was defined based on the presence of *MET* amplification (at any reported level) without *MET*ex14 skipping alterations.

The index date was defined as the recorded date of diagnosis of advanced NSCLC. Patients were described at baseline prior to and at their index date, and were followed up after treatment exposure until death, end of the study period, or loss to follow-up to describe effectiveness outcomes.

### 2.2. Data collection and outcomes assessment

Data were extracted from the EMR of each study-eligible patient. MET biomarker data were extracted at any time at or after diagnosis of NSCLC, including test type (liquid or tissue biopsy), test method, laboratory information, test results for the *MET* alteration (including the specific *MET*ex14 alteration, if available), the level of amplification given either by gene copy number (GCN), or the level of amplification indicated in the electronic case report form as positive, strongly positive or very strongly positive. Additionally, any available results for epidermal growth factor receptor (EGFR), anaplastic lymphoma kinase (ALK), Kirsten Rat Sarcoma virus (KRAS), B-Raf proto-oncogene (BRAF), reactive oxygen species 1(ROS1), cell division protein kinase 6 (CDK6) and PD-L1 tests, were extracted. Baseline demographics and clinical characteristics, description of treatment patterns and disease outcomes were also extracted. The performance status of the patients was extracted as Eastern Cooperative Oncology Group Performance Status (ECOG PS), Karnofsky scores (converted into ECOG PS), or from the physician notes stating that the performance status was not impaired.

Each patient’s line of therapy in the advanced setting was determined by two clinical experts independently, then consolidated and compared with lines of therapy assigned by study sites. The line of therapy assessment was based on type of regimen, category of medication, start and end date of drug, treatment length, treatment overlaps, gap in days after treatment end and treatment start, ‘ongoing’ treatment status noted by site during data collection, date of diagnosis of advanced disease, tumor response, date of progressive disease (PD), death, date of death and cause of death for each individual patient.

Patients with a confirmed line of therapy were eligible for treatment pattern analysis and effectiveness outcomes. Similarly, only patients with non-missing information on tumor response or death were included in the outcome analyses. Treatment exposure was categorized into the following: all anticancer therapies, MET inhibitors, chemotherapy only, ICI as monotherapy, ICI as combination therapy, and other therapies.

Best overall response (BOR) was extracted for each patient per line of therapy and classified according to the following Response Evaluation Criteria In Solid Tumors (RECIST) version 1.1-like categories [23]: complete response (CR), partial response (PR), stable disease (SD), PD, and not evaluable. If an extracted response was RECIST-like, it was classified as loosened RECIST in the categories ‘favorable’ or ‘unfavorable’, and undocumented responses were also recorded.

### 2.3. Statistical analysis

The statistical evaluation was performed using the software package Statistical Analysis System v14 or later. Statistical methods included standard descriptive statistics for patient characteristics and biomarker information. Treatment patterns were described in terms of sequence of treatments, number and percentages of anticancer treatment regimens received, by line of therapy from advanced diagnosis through the end of the medical record. Sankey diagrams were used to present treatment patterns over time by line of therapy.

The overall response rate (ORR) to a line of therapy was calculated as the sum of CR + PR for patients under the estimated RECIST v1.1 classification out of all patients with a recorded BOR and displayed with 95% Clopper Pearson confidence intervals (CI). In addition, in a sensitivity analysis the ORR was calculated including loosened RECIST responses (favorable or unfavorable response), and undocumented responses (unknown or not applicable) under the assumption that tumor response evaluations generally are expected to occur every three treatment cycles in clinical practice. Thus, the numerator counted the OR (BOR of CR, PR) as per RECIST v1.1 and patients with favorable response and the denominator counted all patients with a known response (CR, PR, SD, PD, not estimable (NE), other favorable, other unfavorable, and reclassified NE). Remaining undocumented (unknown or not applicable [NA]) responses did not count in the denominator [24]. For the *MET*-amplified cohort, outcome analyses were planned on patients with either mean GCN ≥ 10 or highly amplified by liquid biopsy, or mean GCN ≥ 8 or highly amplified by liquid biopsy.

Overall survival (OS) was reported from the start of a line of therapy after advanced diagnosis, through to the end of the medical record, irrespective of therapy received across different lines of therapies. Due to missing data for progression dates, duration of response (DoR) and progression-free survival (PFS) could not be assessed. Time to next treatment or death (TNTD), used as a proxy of PFS [25,26], was defined as the time from initiation of a therapy to either end of the line of therapy, any next systemic therapy or death as event. TNTD and OS were described using Kaplan–Meier curves and were presented with a summary of associated statistics with 95% CIs.

## 3. Results

### 3.1. Patient characteristics

A total of 117 patients were included in the study from six oncology sites, of which three were located in the USA (Israel n = 18; The Netherlands n = 13, Taiwan, n = 23, USA n = 63). Thirteen patients had information on treatment exposure recorded after the end of the study period (01 January 2010 up until 31 March 2019) but were nevertheless included. The last available date for these patients was on 30 December 2019. In total, 70 patients were in the *MET*ex14 cohort and 47 patients were in the *MET*-amplification cohort.

Patient demographics and clinical characteristics for these patients are presented in Table 1. Patients with *MET*ex14 skipping were, on average, older (median age 74.2 years, Q1-Q3: 66.8; 78.8) than patients with *MET*-amplified NSCLC (median age 63.1 years, Q1-Q3: 55.8; 70.5); there were fewer males (51.4% versus 65.1%) and less white ethnicity (58.6% versus 85.1%). In the *MET*ex14 cohort, nearly half the patients were never smokers (47.1%), whereas 14.9% were never smokers in the *MET*-amplification cohort. The median duration since stopping smoking was 30 years for patients with *MET*ex14 skipping and 6 years for patients with *MET* amplification.

The majority of patients in both cohorts were diagnosed with advanced stage at their initial diagnosis of NSCLC, with adenocarcinoma as the most frequent histology (*MET*ex14: 84.1%, *MET*-amplified: 82.2%). Sarcomatoid carcinoma was only observed in the *MET*ex14 cohort (5.8%). At index date (advanced diagnosis) most patients had Stage IV NSCLC. ECOG PS, Karnofsky score and impaired status were often missing. Almost half of patients had at least one comorbid condition present at the time of initial diagnosis. The most frequently observed comorbidities were uncomplicated diabetes, chronic obstructive pulmonary disease, and peripheral vascular disease. Brain metastases were seen in 19% of patients with *MET*ex14 skipping and 25% of patients with *MET* amplification.

### 3.2. Biomarkers

Biomarker information for both cohorts are presented in Table 2. Determination of *MET*ex14 status (81.4%) and *MET* amplification (76.6%) was mainly performed in tissue biopsy samples. For patients with *MET*ex14 skipping, the most common testing method was next-generation sequencing (NGS) (60.0%), and 8.6% patients had concomitant *MET* amplification. *MET* amplification was detected through fluorescence *in situ* hybridization (FISH) (55.3%) and NGS (44.7%) in the *MET* amplification cohort. The median time from initial diagnosis of NSCLC until a positive *MET* alteration result was 5.6 months (n = 67) in the *MET*ex14 skipping cohort, and <1 month in the *MET* amplification cohort (n = 42).

Few concomitant oncogenic alterations were found in the test results for patients with *MET*ex14 skipping, except for one patient with activating *EGFR* kinase domain mutations (L858R) and two patients with activating *KRAS* mutations in codon 12.

There were four patients with *MET* amplification with a GCN ≥ 10 and five patients with *MET* amplification with a GCN ≥ 8. Patients with *MET* amplification more frequently had biomarker tests for other oncogenic drivers: EGFR (91.5%), ALK (87.2%), KRAS, (80.9%), ROS1 (76.6%), BRAF (59.6%), and CDK6 (29.8%). Compared with patients with *MET*ex14, concomitant alterations were more frequently observed, including five patients with *EGFR* mutations and one patient with *ALK* fusion.

### 3.3. Treatment patterns

**Fig. 1.**
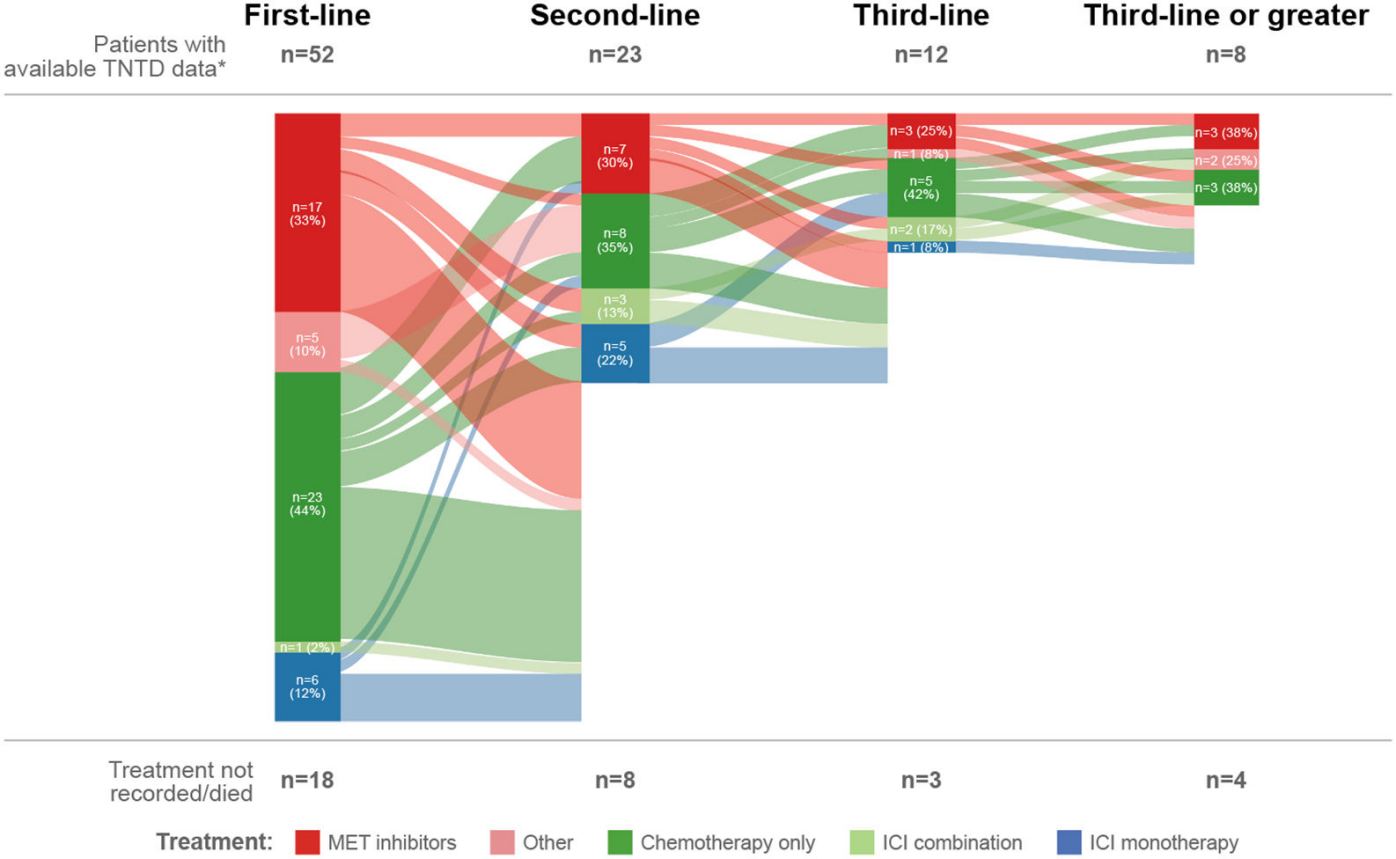
Treatment patterns for the *MET*ex14 skipping cohort within first-, second- and third-line therapy. *N = 70 for patients with *MET*ex14 NSCLC. ICI, immune checkpoint inhibitor; *MET*ex14, *MET* exon 14; NSCLC, non-small cell lung cancer; TNTD, time to next treatment or death.

**Fig. 2.**
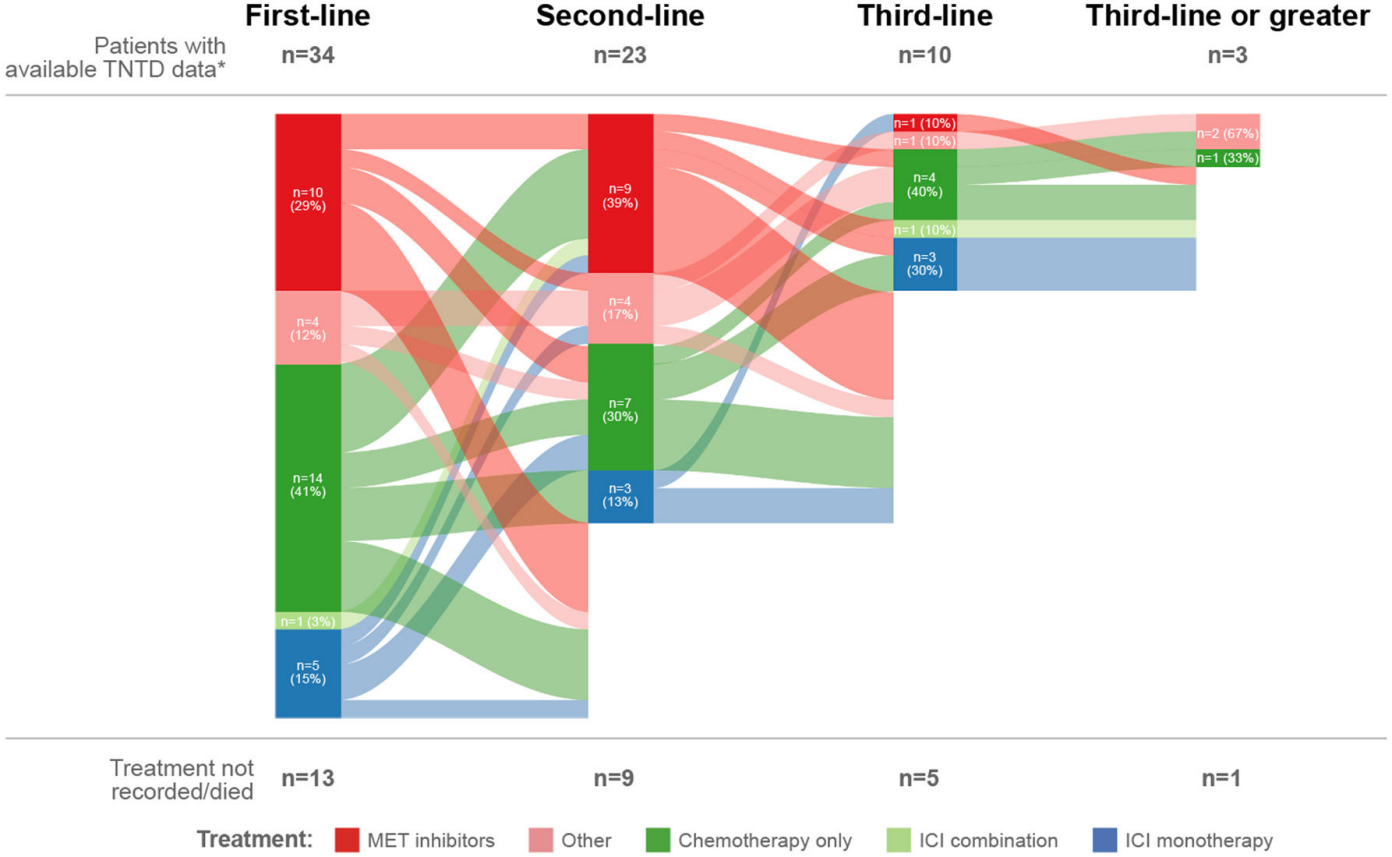
Treatment patterns for the *MET*-amplification cohort within first-, second- and third-line therapy. *N = 47 for patients with *MET*-amplified NSCLC. ICI, immune checkpoint inhibitor; NSCLC, non-small cell lung cancer; TNTD, time to next treatment or death.

Anticancer therapy provided as first-, second- and third-line therapy is displayed by Sankey plots in Fig. 1 (*MET*ex14 cohort) and Fig. 2 (*MET*-amplification cohort) for patients with TNTD data (n = 52 with *MET*ex14 skipping and n = 34 with *MET*-amplification). For both cohorts, the most frequent therapy within first-line therapy was chemotherapy alone (*MET*ex14 cohort 44% [n = 23/24 platinum-based]; *MET*-amplification cohort 41% [n = 14/15 platinum-based]), followed by MET inhibitors (33% [n = 17]; 29% [n = 10]), ICI monotherapy (12% [n = 6]; 15% [n = 5]), and ICI combination therapy (2% [n = 1]; 3% [n = 1]). For patients with *MET*ex14 skipping, chemotherapy remained the most frequent treatment option within second- (35%; n = 1/10 including a taxane) and third-line (42%) therapy, followed by treatment with MET inhibitors (second-line 30%; third-line 25%). MET inhibitors were seen across all lines of therapy for these patients and were primarily given as a monotherapy. In the *MET*ex14 cohort, MET inhibitors comprised crizotinib (n = 17) in first-line; crizotinib (n = 5), capmatinib and cabozantinib (each n = 1) in second-line; and crizotinib (n = 5) and cabozantinib (n = 1) in later-line therapy. For patients with *MET* amplification, treatment with MET inhibitors was most commonly reported in second-line therapy (39%), followed by chemotherapy (30%; n = 2/7 including a taxane). The most frequent third-line therapy was chemotherapy. In the *MET* amplification cohort, MET inhibitors comprised crizotinib (n = 9) and capmatinib (n = 1) in first-line; crizotinib (n = 8) and cabozantinib (n = 1) in second-line; and capmatinib (n = 1) in later-line therapy. Overall, 13/86 patients in first-line, 11/46 patients in second-line and 7/22 patients in third-line received ICI, as monotherapy or in combination.

### 3.4. Effectiveness outcomes

In the *MET*ex14 cohort, 18 patients (first line), 10 patients (second line) and 8 patients (third line) had responses assessed according to RECIST v1.1-like criteria.

**Fig. 3.**
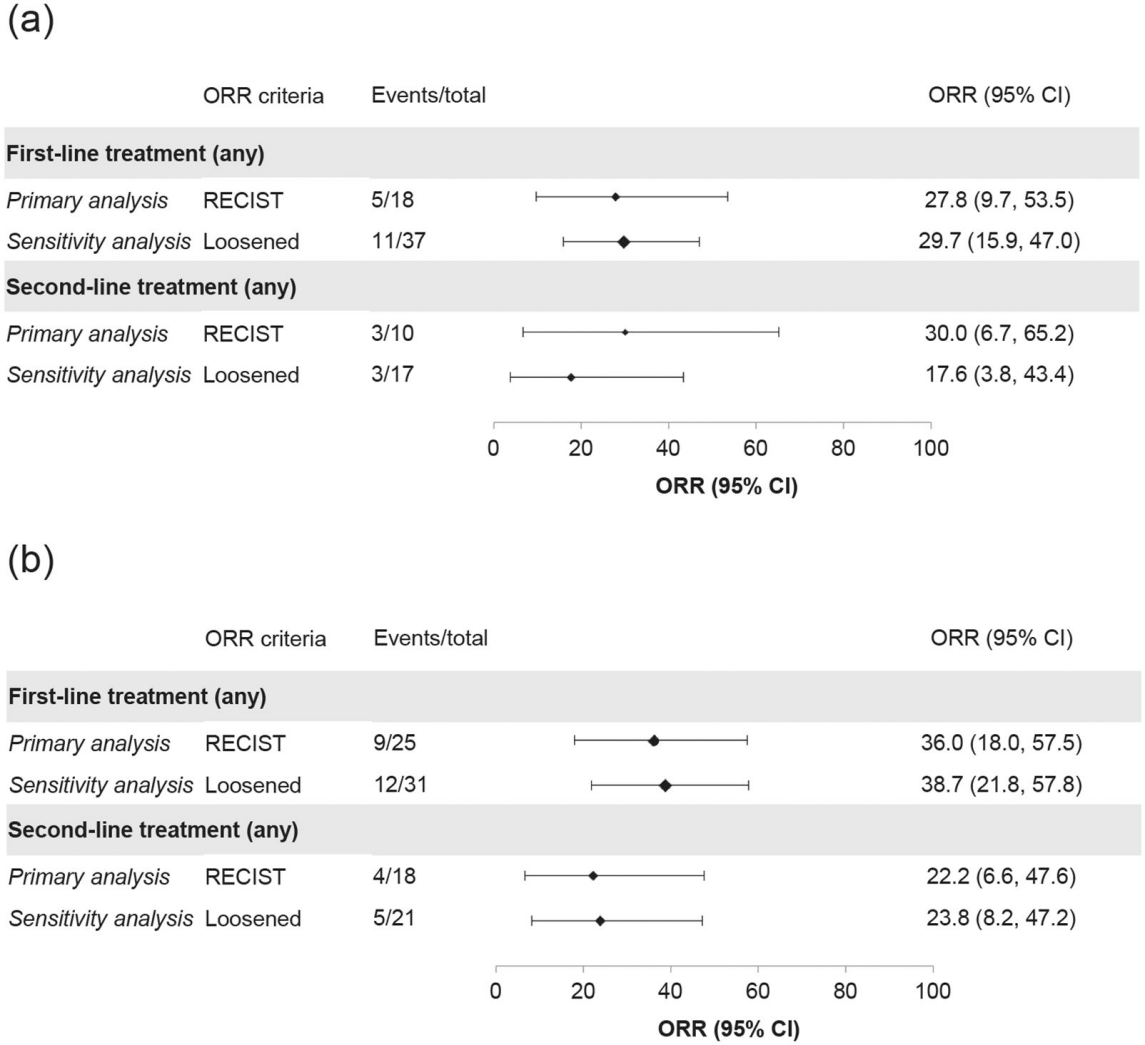
ORR for the first and second line of therapy among patients with *MET*ex14 skipping alterations (a) or *MET* amplification (b). Loosened = ORR was calculated including RECIST responses (favorable or unfavorable response). CI, confidence interval; *MET*ex14, *MET* exon 14; ORR, overall response rate; RECIST, Response Evaluation Criteria In Solid Tumors.

The ORR for the first and second line of therapy among patients with *MET*ex14 skipping alterations is presented in Fig. 3a. For first-line therapy, among patients with response assessed according to RECIST criteria, the ORR was 27.8% (95% CI: 9.7, 53.5), including 40.0% (n = 2/5) in patients who received MET inhibitors and 23.1% (n = 3/13) without MET inhibitors. No responses were observed in patients who received ICI mono or combination therapy (n = 3). For second-line therapy, the ORR by RECIST was 30.0% (95% CI: 6.7, 65.2), including 25.0% (n = 1/4) with MET inhibitors and 33.3% (n = 2/6) without. Again, there were no responses in patients receiving ICI mono or combination therapy (n = 2). In third-line therapy, there were 2/2 responses with MET inhibitors and 0/6 responses without MET inhibitors.

**Fig. 4.**
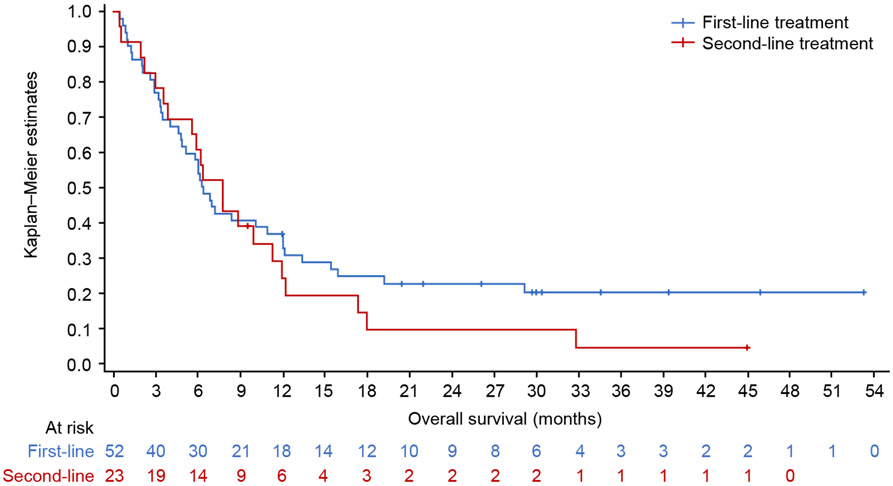
Time to next treatment or death from start of the first or second line of therapy in patients with *MET*ex14 skipping alterations. *MET*ex14, *MET* exon 14.

**Fig. 5.**
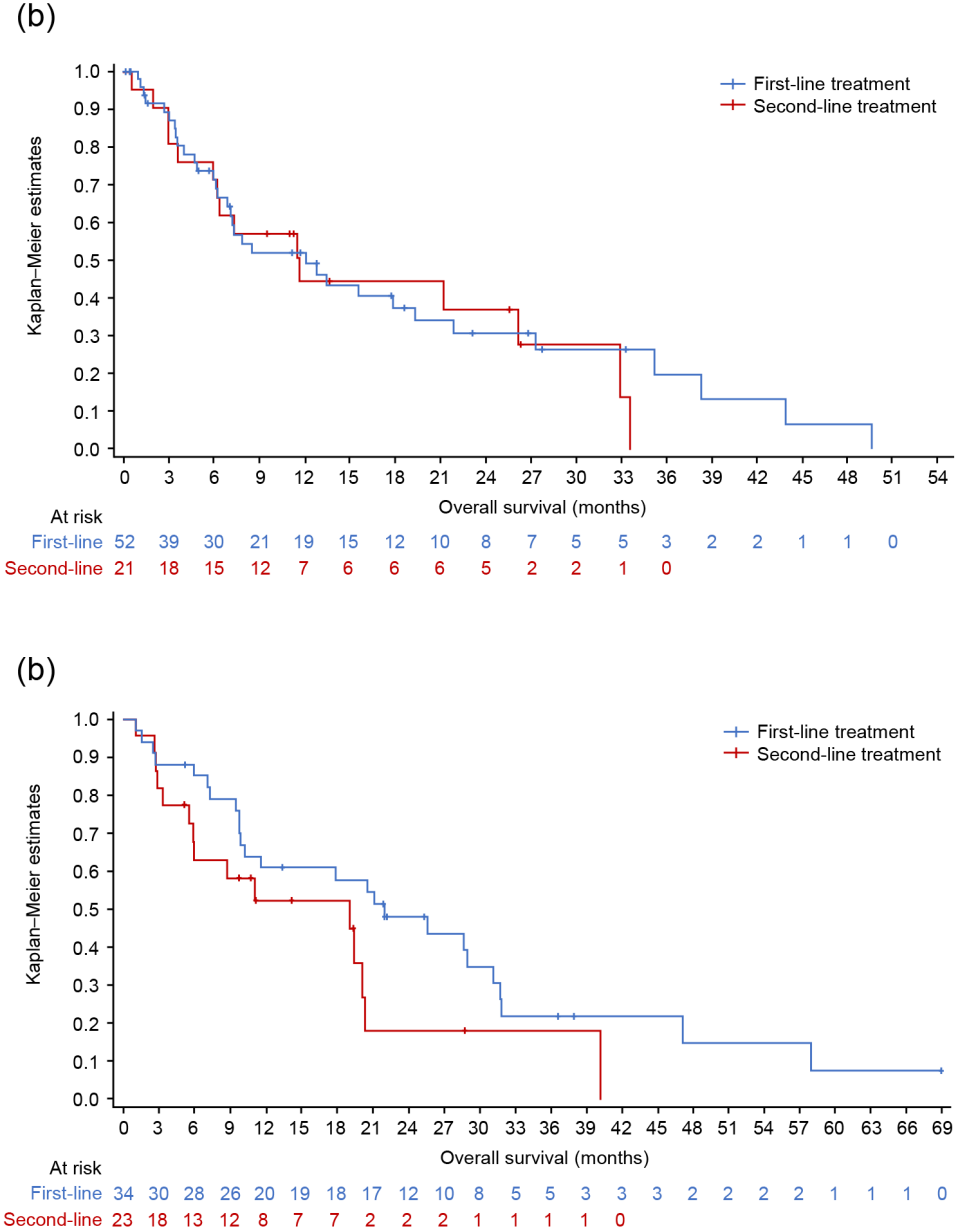
Overall survival from start of first or second line of therapy in patients with *MET*ex14 skipping alterations (a) or *MET* amplification (b). *MET*ex14, *MET* exon 14.

In the *MET*ex14 cohort, the median TNTD was 6.3 months (95% CI: 4.8, 10.9) for first-line therapy (n = 52), and 7.8 months (95% CI: 3.9, 11.3) for second-line therapy (n = 23) (Fig. 4). For patients who received MET inhibitors and those who did not, median TNTD was 10.92 months (95% CI: 3.2, 19.2) and 6.05 months (95% CI: 3.5, 7.2) in first-line, and 10.0 months (95% CI: 2.0, 18.0) and 6.3 months (95% CI: 3.0, 11.3) in second-line, respectively. In the *MET*ex14 cohort, the median OS was 12.0 months (95% CI: 6.8, 19.2) from start of first-line therapy (n = 52), and 11.7 months (95% CI: 6.0, 32.9) from start of second-line therapy (n = 21) (Fig. 5a).

In the *MET*-amplified cohort, 25 patients (first line), 18 patients (second line) and 6 patients (third line) had responses assessed according to RECIST v1.1-like criteria. For first-line therapy, the ORR was 36.0% (95% CI: 18.0, 57.5), including 33.3% (n = 3/9) with MET inhibitors and 37.5% (n = 6/16) without MET inhibitors. There was one CR and one PR among the five patients who receved ICI monotherapy or combination therapy (ORR 40%). For second-line therapy, the ORR was 22.2% (95% CI: 6.4, 47.6) (Fig. 3b), including 42.9% in patients who received MET inhibitors (n = 3/7) and 9.1% in those who did not (n = 1/11). One patient received ICI monotherapy and did not respond. No patients received MET inhibitors in third-line therapy; there were 4/6 responses in patients who did not receive MET inhibitors, including 2/2 responses with ICI. There were insufficient patients numbers (≥10 patients) with information on the level of amplification to perform outcome analyses per level of *MET* amplification. Median TNTD overall was 9.0 months (95% CI: 6.1, 11.7) for first-line therapy (n = 34) and 5.13 months (95% CI: 3.3, 12.8) for second-line therapy (n = 23). For patients who received MET inhibitors and those who did not, median TNTD was 6.88 months (95% CI: 1.4, 11.6) and 9.36 months (95% CI: 4.4, 12.5) in first-line, and 3.09 months (95% CI: 1.1, 20.3) and 8.5 months (95% CI: 4.7, 19.4) in second-line, respectively. In the *MET*-amplified cohort, the median OS was 22.0 months (95% CI: 9.8, 31.2) from start of first-line therapy (n = 34), and 19.0 months (95% CI: 5.5, 20.3) from start of second-line therapy (n = 23) (Fig. 5b).

## 4. Discussion

Our findings present evidence on how the characteristics of patients with *MET*ex14 skipping and *MET*-amplified NSCLC differ, and how the treatment patterns of these patients in the real-world setting are heterogeneous, with non-targeted treatments being commonly used. Adenocarcinoma was the most frequent histology for both cohorts of patients, as has been previously reported, especially for patients harboring *MET*ex14 skipping alterations [4,16,19,27]. However, this finding may have been influenced by changes in clinical practice as, until recently, patients with squamous cell carcinoma histology were not screened for activating mutations. Patients with *MET*ex14 were characterized by a balanced sex ratio (51.4% male) and older age (median 75 years). Advanced age of patients with *MET*ex14 skipping compared to patients with NSCLC, on average, has also been observed in other studies [4,15,16,19,27,28]. This is in contrast to other driver oncogenes in NSCLC, such as *EGFR* and *ALK*, that are known to define a distinct patient population that is typically younger, with a strong female pre-dominance [29-34]. Patients from the *MET*-amplification cohort however, were younger than the typical patient with NSCLC. Similar results were shown in a US retrospective analysis of 99 patients with lung adenocarcinoma, and *MET* amplification (mean *MET* ≥ 5), with a median age of 61 years at diagnosis; 66% were current or former smokers [12]. Similarly, in the *MET*-amplification cohort in the current study, most patients were either current or former smokers and had, on average, only recently stopped smoking. Contrary to this, in the *MET*ex14 cohort, nearly half the patients were never smokers. Over 30% of patients in the *MET*ex14 cohort were Asian, compared with<5% in the *MET* amplification group. While *EGFR* or *ALK* oncogenic alterations are more often reported in Asian patients with NSCLC [35], *MET* alterations such as *MET*ex14 generally appear to be less prevalent in Asian versus Caucasian patients [4,36,37]. The ability of this study to provide additional information on differences in the frequency of oncogenic drivers with different ethnicities is limited. However, while differences in the prevalence of individual oncogenic drivers are observed between populations, the functional impact of oncogenic drivers on NSCLC, i.e. their clinical manifestation and predictability for targeted therapies, is important across populations irrespective of ethnicity. The slightly higher number of Asian patients included in this study provides valuable information on an otherwise under-reported patient population; 23/117 patients in this study were enrolled from a Taiwanese site. In terms of treatment selection, ethnicity is not cited as a relevant factor in the treatment of oncogenic-driven NSCLC in the current Pan-Asian adapted ESMO Clinical Practice Guidelines [11].Until recently, testing for *MET* alterations has not been routine, in part due to the lack of approved treatment options. Accordingly, testing patterns for *MET* alterations were heterogeneous with several testing methods used. NGS was the most frequently used method for *MET*ex14 patients. FISH was most often used to detect *MET* amplification (55.3%) followed by NGS (44.7%). Despite being invasive and possibly inadequate to represent the whole malignancy, tumor tissue biopsy appears to remain the gold standard to investigate potentially actionable biomarkers. Median time to identification for both *MET* alteration types was <6 months from initial diagnosis. In line with other alterations, such as *EGFR* or *ALK*, *MET* alterations are considered to appear early in NSCLC carcinogenesis and do not seem to be affected by other anticancer treatments. Therefore, the time of determination in the clinical course of patients may not be relevant for the effectiveness of MET-directed therapies, although, of course, earlier identification of oncogenic drivers allows for the earlier use of targeted drugs.

The presence of 8.6% concomitant *MET* amplification in the *MET*ex14 cohort is in agreement with previous research; reporting Stage IV *MET*ex14-mutated NSCLC being likely to have some concurrent *MET* amplification [38]. *MET*ex14 skipping is reported to be mutually exclusive with other targetable oncogenic drivers (very few concomitant alterations), whereas the situation is less clear for patients with *MET* amplification where it may depend on the actual copy number increase and focality [39]. In both our study cohorts, patients who were tested seemed to not commonly harbor other oncogenic driver mutations. Interestingly, results from the French Immunotarget study, on 34 patients with NSCLC, showed that PD-L1 was co-expressed with the *MET*ex14 alterations and not with *MET* amplification [40]. Our data suggest that PD-L1 expression occurred at a similar rate in both cohorts.

In both patient cohorts, the treatment landscape was heterogeneous and treatment changes frequent. In the *MET*ex14 cohort, the most common treatment regimen was chemotherapy with or without a vascular endothelial growth factor inhibitor, and MET/HGF inhibitors in both first- and second-line therapy. Similarly, in the *MET*-amplification cohort, the most common types of anticancer therapies were platinum-based chemotherapy regimens and MET/HGF inhibitors. Results reflect changes in the treatment landscape with multiple, recently approved (such as ICIs) and available therapies for patients with NSCLC during the period studied (01 January 2010 up until 30 December 2019).

For patients with *MET*ex14 skipping, the observed ORR assessed according to RECIST v1.1 criteria overall was about 30% for both the first and second lines of therapy. When using loosened tumor response criteria in the first line, results were moderately impacted; however, in the second line, it reduced the observed ORR to about 20%. These results are lower than the ORRs observed in recent trials, investigating the diverse anti-MET tyrosine kinase inhibitors within this specific population (range 32–46%) [41-44], and for patients with *MET*ex14 skipping in line with effectiveness outcome results from a recently similar non-interventional study on a cohort of patients with advanced NSCLC *MET*ex14 [45].

Patients in this study received both standard therapies, such as chemotherapy, and emerging care, such as MET/HGF inhibitors, ICI monotherapy and ICI combination therapy. There is limited published evidence on tumor response across different lines of anticancer therapy in the advanced setting. Sabari and colleagues (2018) reported an ORR of 17% (95% CI: 6.0, 36.0) in an analysis of 24 patients with NSCLC and *MET*ex14 skipping alterations that had received immunotherapy [16]. In first-line therapy, our data showed higher ORR with MET inhibitors (40%) than non-MET inhibitor regimens (23.1%) in patients with *MET*ex14 skipping NSCLC, with the opposite trend in second-line (25% and 33.3%, respectively). In both first- and second-line, median TNTD was approximately 10 months with MET inhibitors and approximately 6 months with non-MET inhibitor regimens. For patients with *MET* amplification, ORR to first-line therapy was similar for MET inhibitors and non-MET inhibitor regimens (33.3% and 37.5%, respectively), and higher for MET inhibitors in second-line (42.9% and 9.1%, respectively) than for regimens without MET inhibitors. However, responses appeared to be less durable with MET inhibitor regimens in first- and second-line (median TNTD 6.9 and 3.1 months, respectively) than for regimens not including MET inhibitors (median TNTD 9.4 and 8.5 months, respectively). These data are, therefore, broadly supportive of MET inhibitor-based therapy versus non-MET inhibitor regimens in patients with *MET*-aberrant NSCLC, although they should be interpreted with caution due to the small sample sizes involved and heterogeneity of the data.

In our study, there were no responses seen in first- or second-line therapy with ICI inhibitors in patients with *MET*ex14 skipping NSCLC, except for one loosened RECIST response observed in the first line. Although these response rates should be interpreted with caution due to small study sample size, they concur with previously observed poor tumor response to ICI among patients with NSCLC with *MET* alterations [40,45,46]. It should also be noted that as the first ICI approval for lung cancer (nivolumab) did not occur until 2014 [47], and our cohort included patients diagnosed between 2010 and 2019, it may be expected that ICI therapy would be, to some extent, under-represented in this analysis compared with the current use of ICIs in NSCLC.

The results from our study are similar compared to the published literature. One study in Korea examining 20 patients with NSCLC and *MET*ex14 skipping, who were observed for first-line therapy, showed a median OS of 9.5 months and a PFS of 4.0 months [21]. Wolf and colleagues (2018) presented results from a multinational, multicenter, retrospective, non-interventional chart review study, and reported on 87 patients with advanced NSCLC with *MET*ex14 mutations; a median survival of 10.9 months (95% CI: 7.4, 16.9) was observed for those who did not receive a MET inhibitor (n = 51), and 25.3 months (95% CI: 18.8, 40.9) for those who did (n = 36) [18]. Similarly, Awad and colleagues (2019) reported on patients with metastatic NSCLC with *MET*ex14 skipping alterations; a median survival of 8.1 months was shown for those who did not receive a MET inhibitor (n = 34), and 24.6 months for those who did (95% CI: 12.1, NA) (n = 27) [19]. The majority of our patients who received a MET inhibitor received crizotinib as either first- or second-line therapy, and the ORRs from our study (27.8% in first-line and 30.0% in second-line) are comparable with the ORR of 32% reported by Drilon et al. [43] in their cohort of 65 response-evaluable patients with *MET*ex14 NSCLC.

In the *MET*-amplification cohort, the median OS in this study is higher than has previously been reported for patients with advanced NSCLC with *MET* amplification. Wolf and colleagues (2018) analyzed patient-level data obtained from a multinational, multicenter, retrospective, non-interventional chart review study on 44 patients with advanced NSCLC with *MET* amplification, and reported a median OS of 17.8 months (95% CI: 7.2, 85.2) in patients who received MET inhibitors (n = 5) and 6.1 months (95% CI: 4.2, 7.9) in those who did not (n = 39). The survival patterns for these patients were, on average, higher than expected considering that the majority of patients were Stage IV at index, and compared to the published literature (although limited). In one recent US cohort study on patients with Stage IV NSCLC, 6,455 patients (66.8%) received first-line therapy, 2,966 (30.7%) received second-line therapy, and 1,204 (12.5%) received third-line therapy. Median OS was 11.1 months (95% CI: 10.8, 11.5) from the index date, 11.7 months (95% CI: 11.3, 12.0) from the initial date of NSCLC diagnosis, and 10.1 months (95% CI: 9.7, 10.4) from the start of first-line therapy. Median OS was longer with first-line immunotherapy (17.5 months [95% CI: 16.9, 18.8]; 60.6% data maturity) versus chemotherapy (15.0 months [95% CI: 14.0, 15.9]; 75.6% data maturity; p < 0.05) and chemotherapy/non-immunotherapy monotherapy (6.8 months [95% CI: 6.6, 7.1]; 77.3% data maturity; p < 0.05). Median OS was 17.5 months (95% CI: 16.8, 18.7; 60.8% data maturity) with first-line chemotherapy and second-line immunotherapy, and was longer compared with first- and second-line chemotherapy (14.2 months [95% CI: 13.6, 14.8]; 76.1% data maturity; p < 0.05) [48].

Censoring assumptions in survival analysis with Kaplan–Meier methods should be ‘non-informative’ – that is, participants who drop out of the study should do so due to reasons unrelated to the study. But, due to the nature of assessments of tumor response in real-world patients, resulting in missing tumor response assessment, we used loosened RECIST criteria as a sensitivity analysis. Certainly, some patients might have missing data or be lost to follow-up for reasons related to the study outcomes. Thus, we think that the missing data, censored in the Kaplan–Meier survival analysis, might be partly informative. Patients who come off study before progression, are likely to have progressed shortly thereafter or died, which reflects not only an increased risk for the censored patients, but a direct dependence on the censoring and progression times. However, the reclassification of patient responses, based on evaluation of treatment patterns and physicians’ assessments, should have minimized the informative censoring.

As this study was based on secondary use of data extracted from patient charts at each participating study site, data entry errors (information bias), while minimized with a standardized data collection process (electronic case report forms and monitoring), at the point of care might still happen but could not be corrected for during analyses. The availability of additional information, such as PD-L1 expression levels and exact timing of diagnostic procedures, could also not be addressed.

The information collected for this study was limited to what was available in the medical records and collected, as part of routine clinical practice but not for research purposes. In that context, a non-interventional study can be used to accurately describe routine clinical practice, and detect associations but cannot establish causality in the same way as prospective, randomized studies unless advanced and robust statistical methods to deal with confounders are applied.

## 5. Conclusions

In patients with both *MET*ex14 skipping alterations and *MET* amplification, the presence of oncogenic drivers other than MET was low. The treatment landscape was diverse. Effectiveness outcomes for non-targeted therapies in this setting indicated that these rare patients have a high unmet medical need and may benefit from a targeted treatment with a MET inhibitor. Nevertheless, the sample size of the study was small, and results could be strengthened with further studies and updated as treatment patterns change in the advanced NSCLC setting.

## Figures and Tables

**Table 1:** Demographics and clinical characteristics of patients with advanced NSCLC and *MET* alterations.

Characteristics	*MET*ex14 cohort (n = 70)	*MET*amp cohort (n = 47)
**Age; n**		
Median (Q1, Q3)	74.2 (66.8, 78.8)	63.1 (55.8, 70.5)
**BMI (kg/m^2^)**	n = 52	n = 29
Median (Q1, Q3)	24.6 (21.7, 27.7)	23.7 (21.4, 28.9)
**Gender, n (%)**		
Male	36 (51.4)	32 (68.1)
Female	34 (48.6)	15 (31.9)
**Race, n (%)**		
White	41 (58.6)	40 (85.1)
Asian	24 (34.3)	2 (4.3)
Black or African American	1 (1.4)	3 (6.4)
Not collected at site or unknown	4 (5.7)	2 (4.3)
**Tobacco smoking status, n (%)**		
Never smoker	33 (47.1)	7 (14.9)
Former or current smoker	34 (48.6)	37 (78.7)
Unknown	3 (4.3)	3 (6.4)
**Time from initial to advanced diagnosis, n (%)**	n = 65	n = 40
Advanced stage at initial diagnosis or < 6 months	55 (84.6)	36 (90.0)
6 months – <1 year	1 (1.5)	1 (2.5)
≥1 year	9 (13.9)	3 (7.5)
**Stage at initial diagnosis of NSCLC, n (%)**	n = 67	n = 45
Stages I–IIIA	12 (18.0)	9 (20.0)
Stages IIIB–IV	55 (82.0)	36 (80.0)
**Histology at initial diagnosis of NSCLC, n (%)**	n = 69	n = 45
Adenocarcinoma	58 (84.1)	37 (82.2)
Sarcomatoid carcinoma	4 (5.8)	1 (2.2)
NSCLC NOS	4 (5.8)	4 (8.9)
Squamous cell carcinoma	1 (1.4)	1 (2.2)
Other	2 (2.9)	1 (2.2)
**Stage of disease of advanced or metastatic NSCLC (study index); n (%)**		
Stage IIIB	9 (12.9)	8 (17.0)
Stage IV	61 (87.1)	39 (83.0)
**Brain metastases; n (%)**	n = 52	n = 28
Present	10 (19.0)	7 (25.0)
**Performance status; n (%)**	n = 38	n = 36
ECOG 0–1*	30 (78.9)	31 (86.1)
ECOG 2–3	8 (21.1)	3 (8.3)
ECOG 4	0	2 (5.6)
**Number of comorbid conditions present at the time of initial diagnosis; n (%)**		
None	31 (44.3)	25 (53.2)
1–3 comorbidities	34 (48.6)	19 (40.4)
>4 comorbidities	5 (7.1)	3 (6.4)
**Most frequent comorbid conditions present at the time of initial diagnosis; n (%)**		
Diabetes uncomplicated	7 (10.0)	3 (6.4)
Chronic obstructive pulmonary disease	6 (8.6)	6 (12.8)
Peripheral vascular disease	6 (8.6)	1 (2.1)
**Past history of neoplasm other than NSCLC; n (%)**		
Yes	6 (8.6)	9 (19.1)

All values are n (%) unless otherwise stated. N numbers are given for where the sample size is less than the total due to missing data.

* ECOG PS 1 was based on Karnofsky assessment for seven *MET*ex14 patients and one *MET* amplified patient. BMI, body mass index; ECOG PS, Eastern Cooperative Oncology Group Performance Status; *MET*amp, *MET* amplification; *MET*ex14, *MET* exon 14; NOS, not otherwise specified; NSCLC, non-small cell lung cancer; Q, quartile.

**Table 2:** Biomarker details for the study cohorts.

Characteristics	*MET*ex14 patients (n = 70)	*MET*amp patients (n = 47)
Type of tumor biopsy test*, n (%)		
Tissue	57 (81.4)	36 (76.6)
Liquid	14 (20.0)	12 (25.5)
**Method of *MET* testing, n (%)**		
Next-generation sequencing	42 (60.0)	21 (44.7)
Real-time polymerase chain reaction	25 (35.7)	0
DNA sequencing	2 (2.9)	0
Polymerase chain reaction	1 (1.4)	0
Fluorescence *in situ* hybridization		26 (55.3)
***MET* amplification (yes), n (%)**	6 (8.6)	47 (100)
**Results of *MET* amplification test, n (%)**		
Positive	–	16 (34.0)
Strongly positive	–	11 (23.4)
Very strongly positive	–	8 (17.0)
Unknown	–	12 (25.5)
***MET* amplification: gene copy number, n (%)**	4 (5.7)	47 (100)
Median (range)	2.8 (0.7 to 5.3)	–
2–8	–	17 (36.2)
8–10	–	1 (2.1)
>10	–	4 (8.5)
Unknown		25 (53.2)
**EGFR test (yes), n (%)**	33 (47.1)^†^	43 (91.5)
Identified alterations	2 (6.1)	5 (11.6)
L858R	1 (3.0)	–
I744F substitution	–	1 (2.3)
T790 mutation	–	3 (7.0)
c.2361G > A	–	1 (2.3)
*EGFR* amplification – equivocal	1 (3.0)	
**KRAS test (yes), n (%)**	22 (31.4)	38 (80.9)
Identified alterations	3 (13.6)	5 (13.2)
D47H	1 (4.5)	–
G12D	1 (4.5)	1 (2.6)
GI2C	1 (4.5)	–
Q61H	–	1 (2.6)
Substitution	–	1 (2.6)
Missense variant – GOF	–	1 (2.6)
**ALK test (yes), n (%)**	29 (41.4)	41 (87.2)
Identified alterations	1 (3.4)	3 (7.3)
EML4-ALK fusion	–	2 (4.9)
ALK L1152V	–	1 (2.4)
H976N	1 (3.4)	–
**ROS proto-oncogene 1 test (yes), n (%)**	22 (31.4)	36 (76.6)
Identified alterations	–	3 (8.3)
Chromosome 6q22	–	2 (5.6)
**PD-L1 test (yes), n (%)**	15 (21.4)	19 (40.4)
PD-L1 expression**	11 (73.3)	8 (42.1)
**CDK6 test (yes), n (%)**	5 (7.1)	14 (29.8)
Identified alterations	1 (20.0)	4 (28.6)
**BRAF test (yes), n (%)**	16 (22.9)	28 (59.6)
Identified alterations	0	4 (14.3)

* Results can be overlapping as patients could test positive by more than one method.

† *EGFR* mutations may also have been included in NGS testing.

** Level of PD-L1 expression: NA.

All values are n (%). N numbers are given for where the sample size is less than the total due to missing data. ALK, anaplastic lymphoma kinase; BRAF, B-Raf proto-oncogene; CDK6, cell division protein kinase 6; EGFR, epidermal growth factor receptor; KRAS, Kirsten Rat Sarcoma virus; *MET*amp, *MET* amplification; *MET*ex14, *MET* exon 14; NA, not available; PD-L1, programmed death-ligand 1; ROS, reactive oxygen species.

## Abbreviations:

ALK: anaplastic lymphoma kinase
BMI: body mass index
BOR: best overall response
BRAF: B-Raf proto-oncogene
CDK6: cell division protein kinase 6
CI: confidence interval
CR: complete response
DoR: duration of response
ECOG PS: Eastern Cooperative Oncology Group Performance Status
EGFR: epidermal growth factor receptor
EMR: electronic medical record
FISH: fluorescence *in situ* hybridization
GCN: gene copy number
HGF: hepatocyte growth factor
ICI: immune checkpoint inhibitor
KRAS: Kirsten Rat Sarcoma virus
MET: mesenchymal epithelial transition factor
*MET*ex14: *MET* exon 14
NA: not available
NE: not estimable
NGS: next-generation sequencing
NSCLC: non-small cell lung cancer
ORR: overall response rate
OS: overall survival
PD: progressive disease
PD-L1: programmed death-ligand 1
PFS: progression-free survival
PR: partial response
RECIST: Response Evaluation Criteria In Solid Tumors
ROS: reactive oxygen species
SD: stable disease
TNTD: time to next treatment or death
USA: United States of America
